# Valosin-Containing Protein (VCP/p97) Is an Activator of Wild-Type Ataxin-3

**DOI:** 10.1371/journal.pone.0043563

**Published:** 2012-09-06

**Authors:** Mário N. Laço, Luisa Cortes, Sue M. Travis, Henry L. Paulson, A. Cristina Rego

**Affiliations:** 1 Center for Neuroscience and Cell Biology, University of Coimbra, Coimbra, Portugal; 2 Faculty of Medicine, University of Coimbra, Coimbra, Portugal; 3 Department of Biochemistry, Carver College of Medicine, University of Iowa, Iowa City, Iowa, United States of America; 4 Department of Neurology, University of Michigan Medical School, Ann Arbor, Michigan, United States of America; Northwestern University, United States of America

## Abstract

Alterations in the ubiquitin-proteasome system (UPS) have been reported in several neurodegenerative disorders characterized by protein misfolding and aggregation, including the polylgutamine diseases. Machado-Joseph disease (MJD) or Spinocerebellar Ataxia type 3 is caused by a polyglutamine-encoding CAG expansion in the *ATXN3* gene, which encodes a 42 kDa deubiquitinating enzyme (DUB), ataxin-3. We investigated ataxin-3 deubiquitinating activity and the functional relevance of ataxin-3 interactions with two proteins previously described to interact with ataxin-3, hHR23A and valosin-containing protein (VCP/p97). We confirmed ataxin-3 affinity for both hHR23A and VCP/p97. hHR23A and ataxin-3 were shown to co-localize in discrete nuclear foci, while VCP/p97 was primarily cytoplasmic. hHR23A and VCP/p97 recombinant proteins were added, separately or together, to normal and expanded ataxin-3 in *in vitro* deubiquitination assays to evaluate their influence on ataxin-3 activity. VCP/p97 was shown to be an activator specifically of wild-type ataxin-3, exhibiting no effect on expanded ataxin-3, In contrast, we observed no significant alterations in ataxin-3 enzyme kinetics or substrate preference in the presence of hHR23A alone or in combination with VCP. Based on our results we propose a model where ataxin-3 normally functions with its interactors to specify the cellular fate of ubiquitinated proteins.

## Introduction

Ataxin-3 is a 42 kDa intracellular protein capable of binding and hydrolysing ubiquitin chains [1]–[3]. Ataxin-3 was first recognized as the protein implicated in Machado-Joseph disease (MJD), also known as spinocerebellar ataxia type 3 [4]. MJD is a polyglutamine disorder, one of a group of nine neurodegenerative diseases that share a common genetic cause: an expansion of a CAG trinucleotide repeat in the coding region of the respective disease genes [5]–[7]. In the case of MJD, the pathogenic expansion occurs in the *ATXN3* gene, which encodes a deubiquitinating enzyme (DUB), ataxin-3. When expanded the CAG repeat encodes an abnormally long polyglutamine (polyQ) track near the C-terminus of ataxin-3 [8]–[9]. The polyQ expansion destabilizes ataxin-3 structure, increasing its propensity to misfold and form large intracellular aggregates [10]–[11]. Through its aggregation, expanded ataxin-3 sequesters house-keeping proteins that are essential for cell homeostasis inside intracellular aggregates [12]–[14]. Although these aggregates were initially speculated to cause neurodegeneration, more recent studies suggest that their formation represents a cellular defense mechanism against smaller toxic oligomers of the mutant protein [15]–[16].

The polyQ expansion alone can not account for all the specific characteristics and selective neuronal loss in MJD. The protein context in which the polyQ is expressed is a crucial determinant of the pathological mechanisms triggered by expanded ataxin-3 [16]–[20]. Many recent studies have focused on wild-type ataxin-3 function. Ataxin-3 has shown to be a DUB with an N-terminal catalytic “Josephin” domain with ubiquitin hydrolase activity, and two or three ubiquitin-interacting motifs (UIM) depending on the splice isoform, responsible for the interaction with ubiquitin chains [1]–[2]. Ataxin-3 possesses higher affinity for longer ubiquitin chains, containing at least four molecules of ubiquitin, and preferentially cleaves linkages between ubiquitin molecules established through lysine 63 (K63) [3]. These characteristics suggest that ataxin-3 is an ubiquitin chain editing enzyme.

As an enzyme, ataxin-3 is regulated at multiple levels in the cell. Mono-ubiquitination of ataxin-3, which is enhanced by proteotoxic stress, increases its ubiquitin hydrolase activity [21]. Ataxin-3's translocation between the nucleus and the cytosol is regulated by nuclear import and export signals in association with specific phosphorylation and dephosphorylation events [22]–[23]. The high degree of regulation sustains a central role for ataxin-3 in the protein quality control of the cell. Ataxin-3 has been implicated in ubiquitin-proteasome pathways [24], endoplasmic reticulum associated degradation (ERAD) [25] and the cytoprotective response to heat shock stress [26].

The study of ataxin-3 enzymatic activity, its nuclear-cytoplasm translocation, and the colocalization of other cellular proteins to intracellular aggregates formed by expanded ataxin-3 have spurred the search for ataxin-3 interactors. A growing number of interactors have been identified that are diverse in structure and function, reflecting the wide range of biological activities associated with ataxin-3 [27]–[30]. Some of these protein interactions occur independently of the polyQ expansion and likely relate to normal ataxin-3 function. In the current study, we have focused on two of these interactors, hHR23A and valosin-containing protein (VCP/p97). hHR23A and hHR23B, the human homologs of RAD23 yeast protein, are involved in DNA repair pathways and the delivery of ubiquitinated substrates to the proteasome for degradation [31]. hHR23A has two ubiquitin-associated (UBA) domains which recognize and bind ubiquitin motifs, and an ubiquitin-like domain which interacts with the globular catalytic domain of ataxin-3 [32]. VCP/p97 protein is a 97 kDa AAA ATPase involved in several cellular pathways, including the extraction of ubiquitinated proteins from the endoplasmic reticulum for degradation by ERAD [25]. VCP/p97 is an abundant cellular protein that has numerous interactions with other proteins [33]. An extensive study has uncovered the region in ataxin-3 crucial to the interaction with VCP/p97, which is situated after the second UIM, just prior to the polyQ domain [28].

In previous reports, ataxin-3 was shown to be a DUB with rather slow kinetics [3]. However, a post-translational modification of ataxin-3, ubiquitination, was highly effective at enhancing ubiquitin hydrolase activity of ataxin-3 [21]. Here, we sought to investigate whether VCP/p97 or hHR23A, both of which are involved in protein degradation pathways, also enhanced ataxin-3 activity through direct interactions. Modulation of ataxin-3 activity through protein-protein interactions might represent an additional level of regulation over its DUB activity and cellular roles. Moreover, a better understanding of how ataxin-3 is regulated may lead to the identification of pathological targets underlying neurodegeneration in MJD.

## Materials and Methods

### Cell line culture

COS 7 cells (ATCC®, UK) were cultured in Dulbecco's modified Eagle's medium (DMEM) supplemented with 10% fetal bovine serum (FBS) (Invitrogen, UK) and 1% streptomycin/penicillin (Gibco, UK), at 37°C and under a 95% air and 5% CO_2_ controlled atmosphere.

### Immunocytochemistry and microscopy

Confluent cultures of COS-7 cells (ATCC®, UK) were split and cells were plated over 13 mm glass coverslips without coating. In the next day, the cells were washed 2 times with phosphate buffered solution (PBS, in mM: 137 NaCl, 2.7 KCl, 1.4 K_2_HPO_4_, 4.3 Na_2_HPO_4_, pH 7.4) before being fixed in 4% paraformaldehyde (PFA) in PBS for 15 minutes. Thereafter, the cells were rinsed 3 times with PBS and opsonized with 5% goat serum in PBT (PBS plus 0.1% Triton X-100) for 1 hour at room temperature. Cells were incubated with primary antibodies: mouse monoclonal anti-ataxin-3 1H9 (1∶1000; Chemicon, USA), rabbit polyclonal anti-hHR23A (1∶50; ProteinTech Group, USA), rabbit polyclonal anti-VCP/p97 (1∶200; Cell Signaling, USA) diluted in 5% goat serum in PBT for 1 hour at room temperature, then the coverslips were washed 2 times with PBS and immersed in a diluted solution of secondary antibodies: anti-mouse Alexa-fluor 594 (1∶250; Molecular Probes, USA) and anti-rabbit Alexa-fluor 488 (1∶250; Molecular Probes, USA) in 5% goat serum in PBT for another hour at room temperature. Finally, cells were stained with Hoechst 33342 (1 µg/µl; Molecular Probes, USA) in PBS for 5 minutes, before mounting the coverslips in DAKO solution. Confocal images were taken in a LSM 510 Meta confocal microscope (Carl Zeiss, Germany) with a EC-PlanNeofluar (40× magnification) and a Plan-ApoChromat (63× magnification) using a Diode 405-30 (405 nm), Argon/2 (488 nm) and DPSS 561-10 (561 nm) lasers.

### Plasmids

The construction of pGEX-6P1 vectors expressing wild-type (Q22) ataxin-3, catalytically inactive (C14A) ataxin-3 mutant and expanded (Q80) ataxin-3 was previously described [3]. hHR23A and human VCP/p97 plasmids were generated by digesting the corresponding pcDNA3-hHR23A and pET28-VCP/p97-wt with BamHI and NotI, followed by insertion of the released fragments into pGEX-6P1 vector. ^282^RKRR-HNHH (Q22) and (Q80) ataxin-3 constructs were generated through mutation of the respective pGEX-6P1-(Q22), -(Q80) ataxin-3 plasmids with forward primer 5′-CTTACTTCAGAAGAGCTTCATAACCATCA and reverse primer 5′-GCTGCTGTTTTTCAAAGTAGGCTTCATGA by polymerase chain reaction (PCR).

### Recombinant protein purification

Glutathione-S-transferase (GST) fusion proteins were purified as previously described [34]. Briefly, pGEX-6P1 plasmids encoding GST-Atx-3 (Q22), GST-Atx-3 (Q22) ^282^RKRR-HNHH, GST-Atx-3 (Q80), GST-Atx-3 (Q80) ^282^RKRR-HNHH, GST-hHR23A and GST-VCP/p97 were transformed into BL21 E. coli cells (GE Healthcare, UK) and individual colonies were obtained by selective growth in solid Luria Bertani medium (LB) plus ampicillin at 37°C overnight. In the following day, 1 ml of the overnight growth was used to inoculate 100 ml of liquid LB for additional growth at 37°C until reaching an optical density = 0.4. GST-fusion proteins expression was induced with 1 mM isopropyl-1-thio-D-galactopyranoside for 5 h at 30°C. After this expression period, the cells were centrifuged at 4000 rpm for 15 min at 4°C and resuspended in ice-cold lysis buffer (150 mM NaCl, 50 mM NaH_2_PO_4_, 10% glycerol, 0.5 mM ethylenediamine tetraacetic acid (EDTA), 1 mM 1,4-dithiothreitol (DTT)), sonicated, and centrifuged for an additional 10 min to remove debris. The supernatants were incubated with glutathione-Sepharose beads (GE Healthcare, UK) in ice, for 30 min, with gentle agitation and the beads were rinsed 4 times with PreScission protease cleavage buffer (50 mM Tris-HCl, 150 mM NaCl, 10 mM EDTA, 1 mM DTT, pH 8.0, 20% glycerol), before being incubated with PreScission protease (80 units/ml, GW Healthcare, UK) in cleavage buffer overnight, with gentle agitation. The next day, beads were centrifuged in Spin-X centrifuge tube filter columns (Corning Costar, USA) and the supernatant containing the recombinant protein free of the GST domain was collected and stored at −80°C.

### Immunoprecipitation

For the immunoprecipitation of endogenous proteins, COS-7 cells (ATCC®, UK) were lysed in FLAG lysis buffer (50 mM Tris, 150 mM NaCl, 1 mM EDTA, 1% Triton X-100, pH 7.4) supplemented with 1 µg/ml protease inhibitor cocktail (chymostatin, pepstatin A, leupeptin, and antipain). For the immunoprecipitation from *in vitro* samples, 100 nM of both recombinant proteins were incubated in deubiquitination buffer (50 mM HEPES, 0.5 mM EDTA, 0.1 µg/µl ovalbumine , 1 mM DTT, 1 µg/ml protease inhibitor cocktail, at 37° C for 5 hours. Cell lysates and *in vitro* samples were washed with Protein A Sepharose beads (GE Healthcare, UK) to reduce nonspecific binding to the beads, before the incubation with mouse monoclonal (1H9; 1∶1000) or rabbit polyclonal (MJD; 1∶1000) anti-ataxin-3 antibodies overnight, at 4°C with gentle agitation. Beads were then precipitated by centrifugation at 500× g, 4°C for 5 min, washed six times with FLAG lysis buffer, and the protein was eluted with Laemmli buffer at room temperature. The eluted immunoprecipitates were collected in Spin-X centrifuge tube filter columns by centrifugation and stored at −80°C and resolved by SDS-PAGE followed by western blotting.

### Pull-down experiments

Recombinant GST-VCP/p97 fusion protein or GST protein alone were expressed in BL-21 cells (GE Healthcare, UK) as described in the recombinant protein purification section. The cells were sonicated and centrifuged to discard cell debris. The lysates were poured into columns containing glutathione-Sepharose beads (GE Healthcare, UK) and incubated for 30 min on ice with occasional agitation. Beads were washed 6 times with FLAG lysis buffer before being added to COS-7 total extracts in FLAG Lysis buffer, which had been previously incubated with glutathione beads for 1 hour to reduce unspecific interactions with the beads. COS-7 total extracts were in contact with the beads for 2 hours, at 4°C in constant agitation. Beads were washed another 4 times with FLAG lysis buffer supplemented with protease inhibitors and resuspended in Laemmli buffer for 15 min, at room temperature. Beads were centrifuged a last time for 5 min, at 500× g and the elutants were stored at −80°C.

### Protease assays

100 nM human recombinant wild-type (Q22) or expanded (Q80) ataxin-3 was incubated with 250 nM K63 or K48 linked hexa-ubiquitin chains (Boston Biochem, USA) in the absence or presence of 100 nM recombinant VCP, 100 nM recombinant hHR23A, or both. The protease assay was performed in *in vitro* deubiquitination buffer, at 37°C for up to 20 hours. Samples from the reaction mixture were collected at 0, 2, 5 and 20 hours. Reactions were stopped with loading buffer at room temperature.

### SDS-PAGE and immunoblotting

Total extracts from COS-7 cells (ATCC®, UK), ubiquitin protease assay and immunoprecipitation samples were collected and separated by SDS-PAGE using 10% polyacrylamide gels. Afterwards, the proteins were transferred onto polyvinylidene difluoride membranes (Hybond-P, GE Healthcare, UK) which were further blocked in a 5% non-fat milk solution for 60 minutes. Membranes were incubated with a mouse monoclonal anti-ataxin-3 (1H9, 1∶1000, Chemicon), a rabbit polyclonal anti-hHR23A (1∶1000; ProteinTech Group, USA), a rabbit polyclonal anti-VCP/p97 (1∶1000, Cell Signaling, USA) or a rabbit polyclonal anti-ubiquitin (1∶1000, Dako, Denmark) antibodies overnight, at 4°C with agitation. After being washed, the blots were incubated with alcaline phosphatase conjugated anti-mouse secondary antibody (GE Healthcare, UK) and developed with enhanced chemifluorecence. The membranes were visualized in a Biorad VersaDoc Imaging System Model 3000.

### Statistical analysis

Data were expressed as mean ± SEM of the number of experiments indicated in the figure legends. Comparisons between two conditions were performed by two-tailed unpaired t-test. Significance was defined at p<0.05.

## Results

### VCP/p97 and hHR23A interact directly with ataxin-3

It was previously shown that ataxin-3 can bind and cleave ubiquitin chains [1]–[3]. Since protein-protein interactions are known to modulate enzymatic activity, we hypothesized that hHR23A and/or VCP/p97 could modify the ubiquitin hydrolase activity of ataxin-3 through direct interaction. To test this hypothesis we investigated the interaction between these three proteins *in vitro* and in cells.

**Figure 1:**
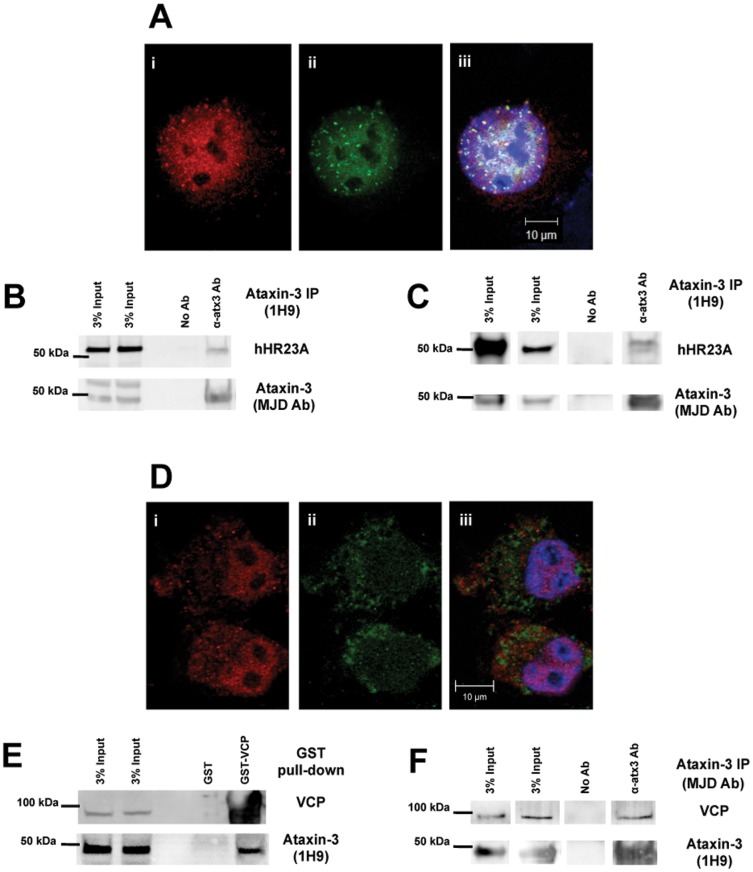
Ataxin-3 interacts directly with hHR23A and VCP/p97. (**A**) Confocal microscopy images of COS-7 cells fixed and immunostained for endogenous ataxin-3 (i-red) and hHR23A (ii-green). DNA was stained with Hoescht 33342 (blue) in the merged image (iii). (**B**) Endogenous ataxin-3 was immunoprecipitated from COS-7 cell extracts and co-immunoprecipitation of endogenous hHR23A was determined by western blotting. (**C**) Recombinant human ataxin-3 and recombinant human hHR23A were incubated under the same conditions applied in the *in vitro* protease assays, for 5 hours at 37°C. Recombinant ataxin-3 was precipitated using anti-ataxin-3 (1H9) antibody and hHR23A co-immunoprecipitation was analyzed by western blotting. (**D**) Visualization of endogenous ataxin-3 (i-red) and VCP/p97 (ii-green) through confocal microscopy in fixed COS-7 cells using specific antibodies. Nuclei were stained Hoescht 33342 dye (blue) for merged images. (**E**) Recombinant GST-VCP/p97 fusion protein was used to pull-down endogenous ataxin-3 from COS-7 total extracts. Endogenous ataxin-3 and recombinant VCP/p97 were detected by western blotting. (**F**) Recombinant human VCP/p97 and recombinant human (Q22) ataxin-3 were added to a buffered solution at 37°C for 5 hours. These samples were used to immunoprecipitate ataxin-3 and co-immunoprecipitation of VCP/p97 was assessed by western blotting.

Immunostaining for hHR23A and ataxin-3 in COS-7 cells revealed a strong spatial correlation (Figure 1A). Endogenous hHR23A and ataxin-3 were both more prevalent in the nucleus. Within this organelle, hHR23A and ataxin-3 exhibited a punctate pattern with additional diffuse distribution throughout the nucleoplasm, excluding the nucleolus. Ataxin-3 and hHR23A were often present in the same nuclear puncta, although the proteins did not perfectly co-localized through the entire cell. The presence of both proteins in specific subnuclear regions may indicate the compartmentalization of nuclear areas dedicated to protein degradation. Immunoprecipitation of endogenous ataxin-3 from COS-7 cell extracts co-precipitated endogenous hHR23A, suggesting that these co-localized proteins also interact in the cell (Figure 1B). As previously reported [27], [35], immunoprecipitation of recombinant ataxin-3 co-precipitated recombinant hHR23A, demonstrating that hHR23A and ataxin-3 directly interact *in vitro* (Figure 1C).

VCP/p97 is an abundant cellular protein that is homogeneous distributed throughout the cytosol of immunostained COS-7 cells, as shown in Figure 1D. Unlike ataxin-3 and hHR23A, VCP/p97 was not enriched in the nucleus. Nevertheless, recombinant GST-VCP/p97 fusion protein bound to GSH-beads was able to pull-down endogenous ataxin-3 from COS-7 cell extracts (Figure 1E), indicating a significant interaction between the two proteins. Recombinant human wild-type (Q22) ataxin-3 also co-immunoprecipitated recombinant VCP/p97 *in vitro* (Figure 1F), supporting a direct interaction between ataxin-3 and VCP/p97.

### VCP/p97 enhances wild-type ataxin-3 activity *in vitro*

**Figure 2:**
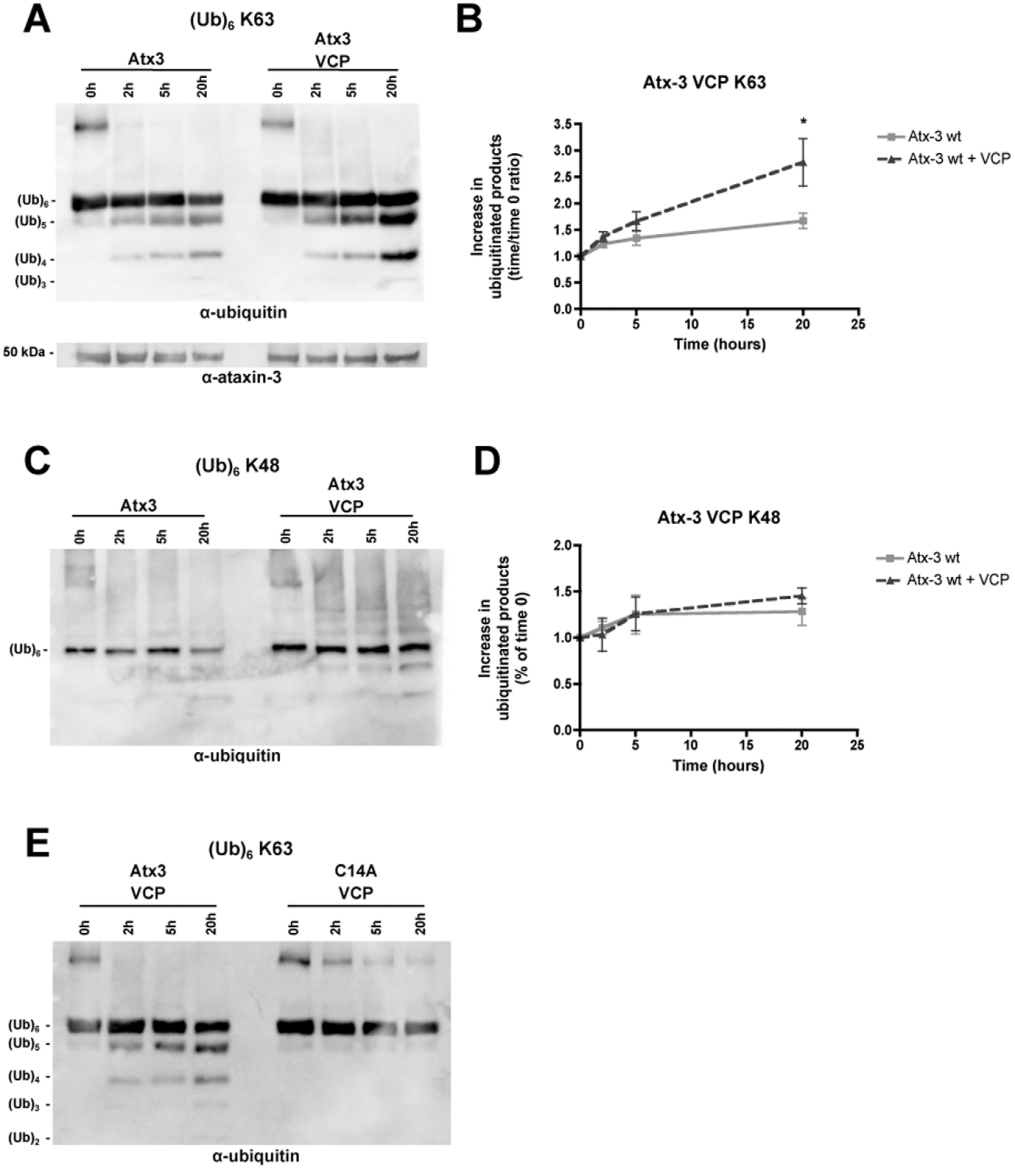
VCP/p97 enhances deubiquitinase activity of ataxin-3 *in vitro*. K63-linked (**A**) and K48-linked (**C**) hexa-ubiquitin chains (250 nM) were incubated with recombinant human (Q22) ataxin-3 (100 nM) with or without recombinant human VCP/p97 (100 nM) for 20 hours at 37°C. Samples collected at 0, 2, 5 and 20 hours were submitted to SDS-PAGE analysis and reaction products were detected by immunoblotting with an anti-ubiquitin antibody. The graphs summarize the mean ± SEM of the increase in reaction products over time resulting from the hydrolysis of K63-linked (**B**) and K48-linked (**D**) hexa-ubiquitin chains by ataxin-3 in the absence (light grey squares) or presence (dark grey triangles) of VCP/p97 in 3–5 independent experiments. Statistical analysis: * p<0.05, compared to recombinant human wild-type ataxin-3 alone at 20 hours. (**E**) K63-linked hexa-ubiquitin chains (250 nM) were incubated with recombinant wild-type or catalytically inactive (C14A) ataxin-3 (100 nM) in the presence of VCP/p97 (100 nM) for 20 hours at 37°C. The 0, 2, 5 and 20 hours time-points were analysed by western blotting with an anti-ubiquitin antibody.

Ataxin-3 interacts with VCP/p97 through a region near the polyQ domain and far from the Josephin domain [28]. The interaction with VCP/p97 may position ataxin-3 in close proximity to polyubiquitinated protein substrates in the cell, as implied by its regulatory effect of the ERAD pathway [25]. To determine whether VCP/p97 can alter ataxin-3 activity, we performed *in vitro* deubiquitination experiments in which recombinant human VCP/p97 was added to the reaction. Ataxin-3 enzymatic activity was assessed through *in vitro* deubiquitination assays as in Burnett et al. (2003) and Winborn et al. (2008). Ataxin-3 acts on longer ubiquitin chains and exhibits preference for ubiquitin chains with ubiquitin molecules linked through K63 [3]. Thus, an ubiquitin chain containing six ubiquitin molecules linked through K63 was used as an optimal substrate to evaluate the influence of hHR23A in ataxin-3 deubiquitinating activity. Ataxin-3 (Q22) incubated with K63 linked hexa-ubiquitin chains generated lower molecular weight reaction products, resulting from the cleavage of ubiquitin linkages (Figure 2A). When VCP/p97 was added to the reaction employing K63-linked chain, an increase in the accumulation of reaction products was observed (Figures 2A, 2B). In contrast, VCP/p97 did not enhance significantly the cleavage of K48-linked chains by ataxin-3 (Figures 2C, 2D). Increased cleavage of K63-linked chains in the presence of VCP was not due to ataxin-3 stabilization, because there were no differences in ataxin-3 levels through the reaction course, in the presence or absence of VCP (Figure 2A). To rule out the possibility that the enhanced K63-linked chain cleavage reflected a direct activity of VCP/p97 on ubiquitin chains, we incubated catalytically inactive ataxin-3 mutant (C14A) with VCP/p97 under the same reaction conditions. As shown in Figure 2E, no reaction products were detected, demonstrating that VCP/p97 enhances ataxin-3 enzymatic activity rather than act directly on ubiquitin chains.

### VCP/p97 does not enhance ubiquitin hydrolase activity of expanded ataxin-3

**Figure 3:**
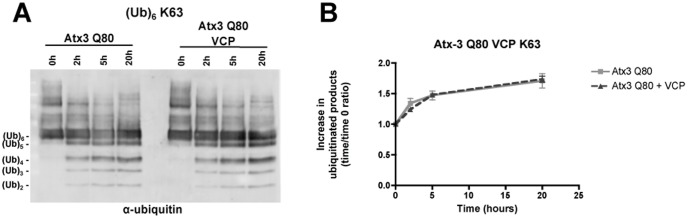
VCP/p97 does not enhance expanded ataxin-3 activity in vitro. (**A**) Recombinant human expanded (Q80) ataxin-3 (100 nM) was incubated with K63-linked hexa-ubiquitin chains (250 nM) in the absence or presence of recombinant human VCP/p97 (100 nM), for 20 hours at 37°C. Samples of 0, 2, 5 and 20 hours time-points were separated by SDS-PAGE and immunoblotted for ubiquitin. (**B**) The graph shows the mean ± SEM of the increase in reaction products over time resulting from the deubiquitinase activity of expanded ataxin-3 over K63-linked hexa-ubiquitin chains in the absence (light grey squares) or presence (dark grey triangles) of VCP/p97 in 4 independent experiments.

Previous *in vitro* studies of ataxin-3 activity have not identified any differences in substrate preference or reaction kinetics between wild-type (Q22) and expanded (Q80) ataxin-3 [3]. Nevertheless, we sought to investigate whether VCP/p97 similarly stimulated the protease activity of expanded ataxin-3. To test this, we expressed and purified human recombinant ataxin-3 with 80 glutamines (Q80). We assayed recombinant human expanded ataxin-3 (Q80) in the same reaction conditions used for nonpathogenic ataxin-3, in the absence or presence of VCP/p97. Unexpectedly, VCP/p97 did not alter expanded ataxin-3 activity towards K63-linked ubiquitin chains (Figures 3A, 3B). This result validates the direct VCP stimulation of wild-type ataxin-3. An indirect effect of VCP/p97 over ataxin-3 activity (e.g. increased accessibility to the ubiquitin chains) would produce a similar effect in both wild-type and expanded proteins.

### Blockade of VCP/p97-ataxin-3 protein interaction abolishes VCP/p97 stimulation of ataxin-3 protease activity

**Figure 4:**
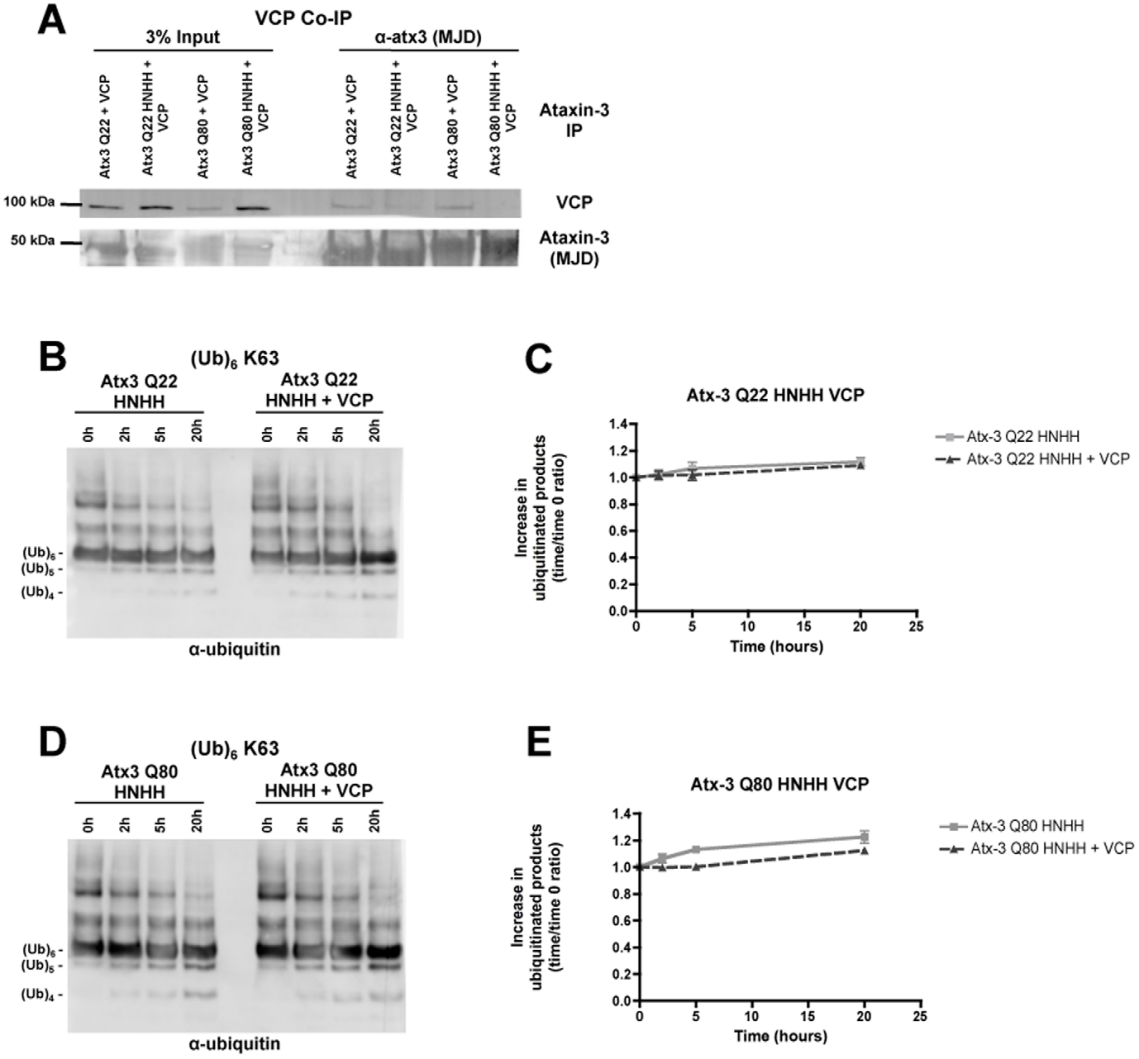
VCP/p97 enhancement of wild-type ataxin-3 activity is mediated through a direct protein-protein interaction. (**A**) Co-immunoprecipitation of recombinant human VCP/p97 (100 nM) with ataxin-3 (100 nM) with 22 or 80 glutamines in its wild-type or (^282^RKRR-HNHH) mutant form, after a pre-incubation of 5 hours at 37°C in a buffered solution. Ataxin-3 forms were precipitated with an anti-ataxin-3 polyclonal antibody (αMJD) and co-immunoprecipitation was assessed by western blotting using anti-VCP/p97 polyclonal antibody. (**B**) Ubiquitin protease assay for Q22 and Q80 (^282^RKRR-HNHH) ataxin-3 mutants (100 nM) in the absence or presence of recombinant human VCP/p97 (100 nM), using K63-linked hexa-ubiquitin chains (250 nM) as substrate. Samples were collected at times 0, 2, 5 and 20 hours and analyzed by western blotting using an anti-ubiquitin antibody. Graphs express the mean ± SEM of the increase in reaction products over time resulting from the hydrolysis of K63-linked hexa-ubiquitin chains by Q22 (**C**) and Q80 (**D**) (^282^RKRR-HNHH) ataxin-3 mutants in the absence (light grey squares) or presence (dark grey triangles) of VCP/p97 from 3 independent experiments.

The region of ataxin-3 that mediates the interaction with VCP/p97 is located between the first two ubiquitin-interacting motifs (UIM)s, near the polyQ track [28]. A sequence of four amino acids in ataxin-3 is crucial for this interaction, the basic amino acids ^282^RKRR (arginine/lysine-rich motif), which also constitute part of a nuclear localization signal [22], [36]–[37]. A four amino acid substitution (^282^RKRR to HNHH) has been shown to block the interaction between ataxin-3 and VCP/p97 *in vitro*. We substituted these four amino acids in wild-type (Q22) and expanded (Q80) ataxin-3 constructs in order to block the interaction between ataxin-3 and VCP/p97. Recombinant ataxin-3 mutants were incubated with human recombinant VCP/p97 under the same experimental conditions of *in vitro* deubiquitination assays, followed by immunoprecipitation of ataxin-3. As expected, the ^282^RKRR - HNHH amino acids substitution effectively reduced the interaction of ataxin-3 mutants with VCP/p97 *in vitro* (Figure 4A). Whereas VCP/p97 was co-immunoprecipitated with wild-type (Q22) and expanded (Q80) ataxin-3, almost no VCP/p97 co-immunoprecipitated with the wild-type (Q22) and expanded (Q80) (^282^RKRR-HNHH ataxin-3 variants). We then tested the ubiquitin hydrolase activity of the ^282^RKRR-HNHH ataxin-3 mutants *in vitro*. Wild-type (Q22) and expanded (Q80) ^282^RKRR-HNHH mutants were both catalytically active, however the protease activity of these mutants was slightly reduced compared to normal Q22 and Q80 ataxin-3 (Figure S1), registering a decrease in the total amount and in the rate of production of low molecular weight ubiquitin chains (Figures 4B, 4D). Moreover, adding VCP/p97 to the reaction system did not increase the ubiquitin hydrolase activity of either ^282^RKRR-HNHH mutant (Figures 4C, 4E). These results demonstrate that VCP/p97 stimulation of wild-type (Q22) ataxin-3 protease activity is mediated through a direct interaction with the enzyme.

### hHR23A does not change the kinetics or the substrate preference of ataxin-3

**Figure 5:**
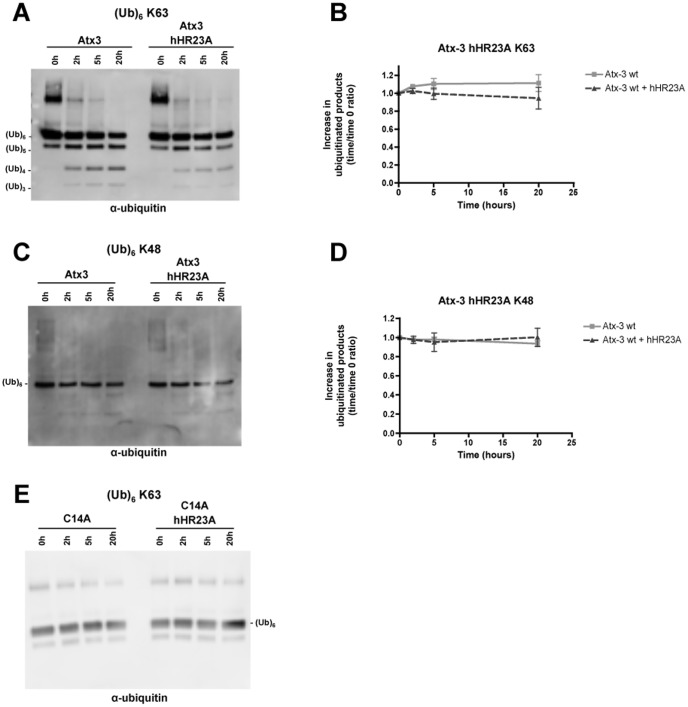
hHR23A has no effect over the ubiquitin hydrolase activity of ataxin-3. Recombinant human (Q22) wild-type (**A, C**) or catalytic inactive (C14A) (**E**) ataxin-3 (100 nM) was incubated alone or together with recombinant hHR23A (100 nM) in a buffered solution containing hexa-ubiquitin chains (250 nM) with K63 (**A, E**) or K48 (**C**) linkages for 20 hours at 37°C. Samples were collected at 0, 2, 5 and 20 hours and analyzed by immunoblotting. The reaction products were detected using an antibody against ubiquitin (**A, C, E**). The appearance of reaction products resulting from the cleavage of K63-linked (**B**) or K48-linked (**D**) hexa-ubiquitin chains by ataxin-3 in the absence (light grey squares) or presence (dark grey triangles) of hHR23A was quantified; the mean ± SEM of 4 independent experiments was plotted in graphs.

hHR23A can simultaneously bind ubiquitin chains and ataxin-3 [27], [32], [38]. In our co-localization studies, hHR23A and ataxin-3 revealed a high homology in their nuclear distributions. For these reasons, hHR23A emerged as a strong candidate for a direct modulator of the ubiquitin hydrolase activity of ataxin-3. To test this model, we quantified the enzymatic activity of recombinant human wild-type ataxin-3 (Q22) in the absence or presence of recombinant hHR23A (Figure 5). As reported previously, ataxin-3 (Q22) deubiquitinates K63-linked ubiquitin chains and promotes the appearance of smaller ubiquitin chains over time. No enhancement in the rate of accumulation of reaction products was observed in the presence of hHR23A (Figures 5A, 5B); rather, there was a trend towards decreased accumulation of reaction products. hHR23A did not alter the substrate preference of ataxin-3 either, as ataxin-3 continued to cleave K63-linked chains more robustly than K48-linked ubiquitin chains (Figures 5C, 5D). Hence, hHR23A does not alter ataxin-3 activity, at least in these *in vitro* assays. Our data is in accordance with a recent report also showing no alteration in the ability of ataxin-3 to cleave ubiquitin chains in the presence of hHR23A [39]. As expected, catalytically inactive ataxin-3 mutant (C14A) failed to generate reaction products (Figure 5E).

### hHR23A blocks the VCP/p97 stimulation of ataxin-3 ubiquitin hydrolase activity

**Figure 6:**
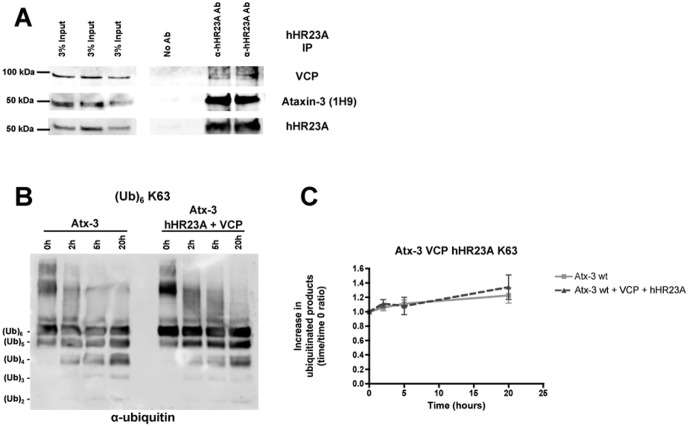
hHR23A blocks the VCP/p97 enhancement of ataxin-3 activity in vitro. (**A**) Endogenous hHR23A was immunoprecipitated from COS-7 cell extracts and the co-immunoprecipitation of endogenous ataxin-3 and endogenous VCP/p97 was determined by western blotting. (**B**) In vitro deubiquitination assay of K63-linked hexa-ubiquitin chains (250 nM) by wild-type (Q22) ataxin-3 (100 nM) alone or in the presence of both hHR23A (100 nM) and VCP/p97 (100 nM). Western blotting was performed for samples collected after 0, 2, 5 and 20 hours of reaction and anti-ubiquitin reactivity was assessed. (**C**) Quantification of the increase in reaction products over time (time = 0 hours as control) resulting from the hydrolysis of K63-linked hexa-ubiquitin chains by wild-type (Q22) ataxin-3 alone (light grey squares) or in the presence (dark grey triangles) of hHR23A and VCP/p97 was plotted as the mean ± SEM of 4 independent experiments.

hHR23A and VCP/p97 are both involved in protein quality control through their connections with protein degradation and the ubiquitin-proteasome system [31]–[33]. Recent studies have established ataxin-3 as an important contributor to ubiquitin-dependent protein quality control [25]–[26]. *Caenorhabditis elegans* homologues for hHR23A, VCP/p97 and ataxin-3 have been previously found to assemble in a trimolecular complex [40]. To assess if these proteins could establish a similar trimolecular complex in our cellular system, endogenous hHR23A was immunoprecipitated from COS-7 cells and co-immunoprecipitation of endogenous VCP/p97 and endogenous ataxin-3 was assessed through western blotting. Accordingly, both endogenous VCP/p97 and endogenous ataxin-3 were co-immunoprecipitated with hHR23A (Figure 6A). Since ataxin-3, hHR23A and VCP/p97 are linked to the same cellular pathways, we assessed whether ataxin-3 enzymatic activity is altered when exposed to two protein interactions with different effects regarding ataxin-3 stimulation. *In vitro* deubiquitination assays were performed with wild-type (Q22) ataxin-3 in the absence or presence of hHR23A and VCP/p97 simultaneously, using K63-linked hexa-ubiquitin chains in the reaction (Figures 6B, 6C). In both conditions wild-type ataxin-3 was able to cleave ubiquitin chains. However, when hHR23A and VCP/p97 were both added to the reaction solution, we did not observe an increase in wild-type ataxin-3 activity (Figures 6B, 6C), as we have previously observed with VCP/p97 alone. Interestingly, ataxin-3 in conjunction with hHR23A and VCP/p97 had similar activity to ataxin-3 alone, displaying neither the increase in activity caused by VCP/p97, nor the trend to reduced activity observed with hHR23A. Taken together, these results suggest that, at least in *in vitro* reactions, hHR23A and VCP/p97 counter the effect of each other on ataxin-3 DUB activity.

## Discussion

In this study, we demonstrate that hHR23A and human VCP/p97 are able to directly interact with human ataxin-3; indeed, we confirm the association of endogenous ataxin-3 with endogenous hHR23A and VCP/p97 [22], [24], [26]–[38]. In our work, VCP/p97 revealed to be a selective activator of wild-type ataxin-3. VCP stimulation of DUB activity was observed for wild-type ataxin-3, but not for the toxic expanded form. These data suggest the existence of a loss of function component on MJD pathology.

Ataxin-3 binds and cleaves poly-ubiquitin chains [1]–[2]. With respect to ubiquitin hydrolase activity, ataxin-3 displays clear substrate preference: ataxin-3 preferentially cleaves K63-linked over K48-linked ubiquitin chains, and longer over shorter chains [3]. Even with its preferred substrates, the reaction kinetics of ataxin-3 *in vitro* are slower than those of other well-studied deubiquitinating enzymes [39]. Recent studies have shown that mono-ubiquitination of ataxin-3 itself is a strong activator of ataxin-3 activity [21], but in cells under basal conditions the amount of mono-ubiquitinated ataxin-3 is minor. Therefore, we investigated whether two well known ataxin-3 interactors could modulate ataxin-3 protease activity.

VCP/p97 is an abundant protein, comprising approximately one percent of total protein in some cells [41]–[42]. Though it is distributed throughout the nucleus and cytoplasm of COS-7 cells, VCP/p97 is more abundant in the cytosol, in contrast with ataxin-3, which is predominantly nuclear in these cells. Despite this difference in localization, we established both a cellular and a direct *in vitro* interaction between ataxin-3 and VCP/p97 in our experiments, confirming previously published results [25], [28], [43]. Importantly, VCP/p97 proved to be an activator of wild-type ataxin-3 ubiquitin hydrolase activity *in vitro*, nearly doubling the accumulation of lower molecular weight ubiquitin chain reaction products. Activation has also been reported for other deubiquitinating enzymes [44]–[45] and most recently, a synergistic cooperation between ataxin-3 and CDC-48 (*C. elegans* orthologue of VCP/p97) was found to have a role in ageing regulation and ubiquitin-mediated proteolysis in *C. elegans* [46]. VCP/p97 did not appear to alter ataxin-3 substrate preference, as K63-linked hexa-ubiquitin chains were still cleaved more extensively than K48-linked hexa-ubiquitin chains. VCP/p97 also had no effect on the activity of mutant (i.e. expanded) ataxin-3. The region of ataxin-3 essential for VCP/p97 interaction is situated just amino-terminal to the polyQ region, so it is conceivable that polyQ repeat length may affect the interaction between the two proteins. Previous studies have reported an increased interaction between VCP/p97 and polyQ-expanded ataxin-3 [28] and our own *in vitro* co-immunoprecipitation studies are consistent with this observation. Even so, this interaction is apparently not sufficient to stimulate the activity of expanded ataxin-3. Although the changes in ataxin-3 structure induced by the expanded polyQ domain seem to promote a stronger association with VCP/p97, the same conformational alterations might abrogate any allosteric effect in favor of catalytic activation. It is important to recall that VCP/p97 normally is organized as a ring-like hexamer rather than as a monomer [47]. A single VCP/p97 hexamer might engage differentially with normal and expanded ataxin-3 polypeptides in a manner that permits activation only of normal ataxin-3 molecules. Additional conformational and structural studies of both proteins in this interaction will be needed to define the mechanism.

Our results confirm that the ^282^RKRR sequence of ataxin-3 mediates binding to VCP/p97 [28] and establish that this region is essential for the activation of ataxin-3 activity by VCP/p97. The ^282^RKRR-HNHH substitution disrupts the interaction of VCP/p97 with wild-type and expanded ataxin-3, and blocks VCP/p97-activation of ataxin-3 DUB activity. We thus conclude that VCP/p97-mediated enhancement of ataxin-3 DUB activity is dependent on direct VCP/p97 interaction with ataxin-3. A competent protein-protein interaction with VCP is mandatory to induce a significant increase in wild-type ataxin-3 activity. Therefore, a more restrained protein-protein interaction might also justify the ineffectiveness of VCP/p97 in increasing expanded ataxin-3 ubiquitin hydrolase activity.

Ataxin-3 resides in the nucleus, cytosol and possibly mitochondria [36], [48]–[49]. In some types of cells, ataxin-3 is predominantly nuclear while in other cells it is largely cytoplasmic. In COS-7 cells, we observed a primarily nuclear distribution of ataxin-3, similar to what we have observed in human fibroblasts and in mouse cerebellar granule cells (Laço et al., unpublished data). Ataxin-3 exhibits the capacity to shuttle in and out of the nucleus, presumably responding to specific stimuli [22], [26], [50]. In the nuclear compartment, ataxin-3 exhibits a punctate pattern with a superimposed, lighter homogeneous distribution throughout the nucleoplasm that spares the nucleolus. This nuclear pattern is very similar to that of hHR23A; indeed, we observed a high degree of co-localization of these two proteins, to the subnuclear foci. Interestingly, the 20S and 19S subunits of the proteasome also exhibit a punctate nuclear distribution in COS-7 cells and several other tested cell types [51]–[52]. These proteasome-enriched nuclear foci are in close association with the ataxin-3 containing-nuclear foci (not shown). The discrete distribution of ataxin-3 and hHR23A into specific nuclear regions, might represent specialized nuclear compartments dedicated to protein quality control and protein degradation.

Interestingly, and despite the tight relation in nuclear distribution and a clear interaction in cellular assays and *in vitro* between ataxin-3 and hHR23A, we found that hHR23A does not influence the ubiquitin hydrolase activity of ataxin-3. hHR23A did not increase ataxin-3 DUB activity or change its substrate preference, in accordance with recent data by Nicastro and colleagues (2010). On the contrary, ataxin-3 actually displayed a non-significant trend towards decreased activity when hHR23A was added to the reaction system. Both ataxin-3 and hHR23A are able to bind ubiquitin chains [53], therefore the decrease in ataxin-3 activity might reflect competition for the ubiquitin chains by the two proteins. Ataxin-3 would be left with less free non- hHR23A bound ubiquitin chains to work on, reducing the quantity of end products of deubiquitination. hHR23A was first proposed to be part of the DNA repair machinery [54]; while other studies have shown an influence of ataxin-3 in nuclear gene expression [29], [55]–[56]. The close association to proteasome-enriched nuclear areas and the influence in expression of specific factors and nuclear proteins levels may link various physiological roles of ataxin-3 and hHR23A to protein degradation.

Recent studies have expanded the number of proteins known to interact with ataxin-3 [29]–[30], [57]–[60]. In a cellular context, ataxin-3 likely does not function alone [25], [61], since protein-protein interactions are established continuously and dynamically in the cell. New protein-protein interactions involving ataxin-3 may be established along with its deubiquitination activity over protein-substrates. Indeed, distinct protein interactions may differently modulate ataxin-3 ubiquitin hydrolase activity, as observed in our study regarding the role of hHR23A and VCP/p97. We exposed ataxin-3 to both hHR23A and VCP/p97 in an attempt to better understand how ataxin-3 activity may be regulated in the cell. Interestingly, the presence of hHR23A negated the activation induced by VCP/p97. Competition between hHR23A and VCP/p97 for a common binding site on ataxin-3 seems unlikely since the described areas of interaction for these two proteins are in different regions of ataxin-3 [28], [38]. Nevertheless, interaction with hHR23A could change the affinity of ataxin-3 for VCP/p97 and thus impair VCP/p97-induced activation of ataxin-3. Also, competition for hexa-ubiquitin chains between hHR23A and ataxin-3 might reduce substrate availability for ataxin-3 when hHR23A and VCP/p97 are added, thereby reducing ataxin-3 DUB activity. On the other hand, ataxin-3 is probably deubiquitinating faster the available ubiquitin chains due to VCP/p97 activation. VCP stimulation of ataxin-3 activity might compensate the lack of substrate and the slight inhibitory effect of hHR23A, originating similar total amounts of reaction products. These interesting data demonstrate that ataxin-3 activity in the cell might be highly regulated and the repercussions of ataxin-3 enzymatic action might derive from the equilibrium of all the various inputs coming from the different interactions ataxin-3 is establishing at a specific moment.

Taken together with previously published studies, our results favor the view that ataxin-3 engages in multiple protein-protein interactions that influence its DUB activity within the cell [25], [30], [40], [59]. Such interactions might influence the selection of ubiquitinated proteins on which ataxin-3 would act. Ataxin-3 is an interesting DUB in part because it can discriminate between different ubiquitin chain conformations [3]. Resembling the phosphorylation and dephosphorylation procedures, different ubiquitin signals made of different ubiquitin chains with different arrangements and topologies will almost certainly trigger different cellular outcomes. The selective activity of ataxin-3 over some ubiquitin linkages may alter the ubiquitin signal on the ubiquitinated substrates and consequently determine their future cellular role. In this way, ataxin-3 may represent an important checkpoint on the future fate of ubiquitinated proteins in the cell and its interactions may play a major role in modulating this event [46].

**Figure 7:**
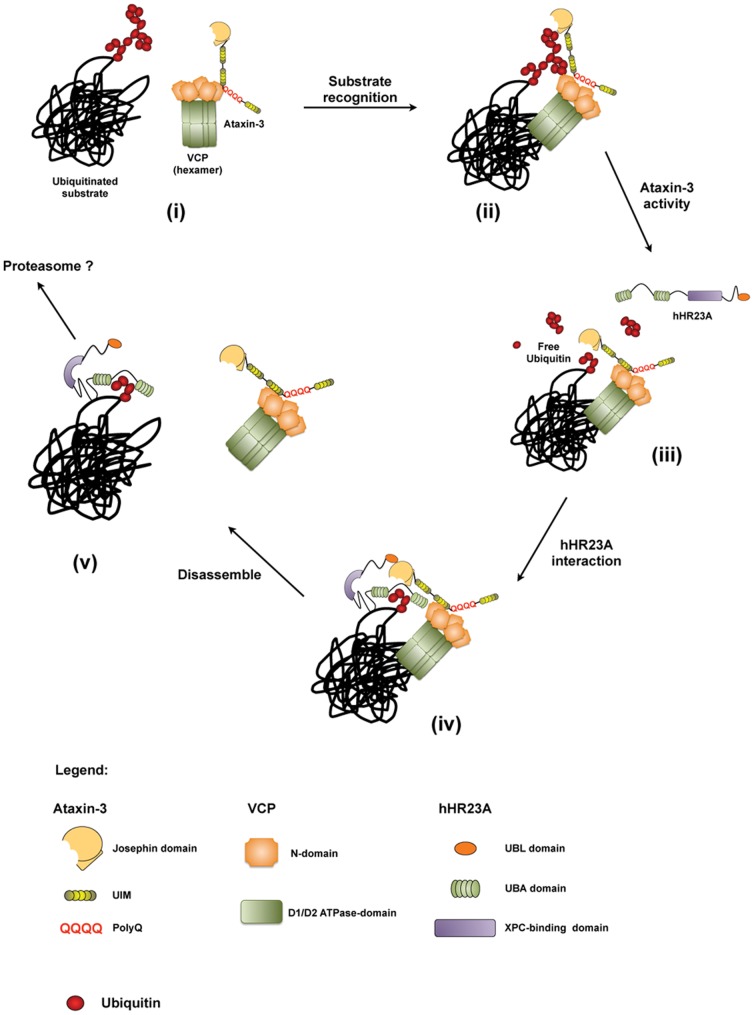
Proposed model of sequential events during the interaction between ataxin-3, VCP/p97 and hHR23A. (i) Ataxin-3 interacts with a monomer of the VCP/p97 hexamer through its ^282^RKRR region, assembling in a complex capable of recognizing the ubiquitinated substrate. (ii) Ataxin-3 acts on the branched ubiquitin chains attached to the ubiquitinated protein, originating a clear and perceptible ubiquitin signal. (iii) Due to its K63-linkage preference, ataxin-3 leaves an all K48-linkage ubiquitin chain on the surface of the protein. (iv) The ubiquitin signal released by ataxin-3 is recognized by hHR23A and interpreted as a degradation signal. (v) hHR23A delivers the ubiquitinated protein to the proteasome for degradation.

Our results and previous published data on VCP/p97 and hHR23A lead us to propose a model in which these proteins interact with ataxin-3 at different points of the processing of ubiquitinated protein-substrates (Figure 7). VCP/p97 and ataxin-3 have been described to work together in the retrotranslocation of ubiquitinated substrates from the endoplasmic reticulum in the ERAD [25]. Recently, it has been shown that ataxin-3 deubiquitinates C-terminus of Hsp70-interacting protein (CHIP) and parkin [3], [59]. VCP/p97 may bind to ubiquitinated CHIP, parkin or other protein-substrates, present them to ataxin-3 and activate the enzyme. Ataxin-3 would cleave preferentially K63 linkages out of the ubiquitin chains, releasing a K48-linkage enriched ubiquitin chain. The interaction with VCP would facilitate the ataxin-3 editing of ubiquitin chains on its substrates. hHR23A would easily recognize the K48-linkage enriched ubiquitin chain left attached to the protein-substrates, since hHR23A has higher affinity for K48 linkages [32], [62]. Finally, hHR23A would take the ubiquitinated substrate out of the catalytic pocket of ataxin-3 and would transfer it to the proteasome or other alternative cellular destination, as depicted in Figure 7.

Taking this model into account, the absence of stimulation of expanded ataxin-3 by VCP/p97 may have strong implications in MJD pathology. We may hypothesize that without the activation of VCP/p97, expanded ataxin-3 would work less efficiently as a DUB, leading to an accumulation of ubiquitinated proteins in the cell. Such a reduction in ubiquitin chain processing by expanded ataxin-3 not only would favor the accumulation, and possibly aggregation, of ubiquitinated proteins in the cell, but also might perturb the delivery of ubiquitinated substrates to their appropriate cellular destination. Thus, the disturbance of normal cellular pathways of ubiquitin signaling would add up to other cellular stresses promoted by the expression of a pathogenic polyQ fragment. The presence of a polyQ expansion in ataxin-3 may impair protein homeostasis through direct inhibition of the proteasome [63]–[64] and thus promote the formation of expanded ataxin-3 aggregates. In addition, the polyQ expansion in ataxin-3 protein may compromise its ubiquitin chain editing activity over ubiquitinated substrates. The buildup of protein malfunction and misleading signals in the cell together with expanded ataxin-3's intrinsic propensity to aggregate, may initiate a chronic cascade of events, culminating in neuronal dysfunction and cell death.

## Supporting Information

Figure S1 **(^282^RKRR-HNHH) ataxin-3 VCP-binding mutants exhibit a small reduction in their protease activity.** Ubiquitin protease assay for (**A**) wild-type (Q22) normal and (^282^RKRR-HNHH) ataxin-3 mutant and (**B**) expanded (Q80) and (^282^RKRR-HNHH) ataxin-3 mutant, using K63-linked hexa-ubiquitin chains as substrate. Samples were collected at times 0, 2, 5 and 20 hours and analyzed by western blotting using an anti-ubiquitin antibody.
(TIF)
