# Supplementary figures and images for: Valosin-Containing Protein (VCP/p97) Is an Activator of Wild-Type Ataxin-3
Source: PLoS One. 2012 Sep 6;7(9):e43563. doi: 10.1371/journal.pone.0043563 (PMC3435318; doi:10.1371/journal.pone.0043563)

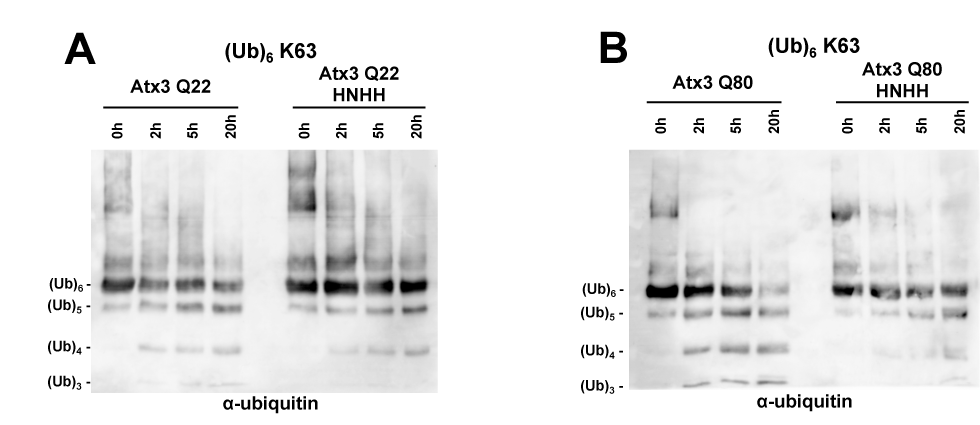

Supplement: Figure S1 — (282RKRR-HNHH) ataxin-3 VCP-binding mutants exhibit a small reduction in their protease activity. Ubiquitin protease assay for (A) wild-type (Q22) normal and (282RKRR-HNHH) ataxin-3 mutant and (B) expanded (Q80) and (282RKRR-HNHH) ataxin-3 mutant, using K63-linked hexa-ubiquitin chains as substrate. Samples were collected at times 0, 2, 5 and 20 hours and analyzed by western blotting using an anti-ubiquitin antibody. (TIF) [file pone.0043563.s001.tif]
